# Willingness to receive intravenous buprenorphine treatment in opioid-dependent people refractory to oral opioid maintenance treatment: results from a community-based survey in France

**DOI:** 10.1186/s13011-017-0131-4

**Published:** 2017-11-02

**Authors:** Perrine Roux, Daniela Rojas Castro, Khadim Ndiaye, Laélia Briand Madrid, Virginie Laporte, Marion Mora, Gwenaelle Maradan, Stéphane Morel, Bruno Spire, Patrizia Carrieri

**Affiliations:** 1 Aix Marseille Univ, INSERM, IRD, SESSTIM, Sciences Economiques and Sociales de la Santé and Traitement de l’Information Médicale, Marseille, France; 2 ORS PACA, Observatoire régional de la santé Provence-Alpes-Côte d’Azur, Marseille, France; 3 AIDES, Poitiers, France; 4 Groupe de Recherche en Psychologie Sociale (EA 4163), Université Lyon 2, Bron, France

**Keywords:** Willingness, Intravenous buprenorphine, Opioid dependence, Cutaneous complications, Community-based research

## Abstract

**Background:**

Injectable opioids are an interesting option for people who inject drugs (PWID) that do not respond to oral Opioid Maintenance Treatment (OMT). To date, intravenous (IV) buprenorphine - a safer drug than full-opioid agonists in terms of overdose risk - has never been tested in a clinical trial on opioid dependence. We designed a survey to better understand the profile of PWID eligible for IV buprenorphine, and their willingness to receive it.

**Methods:**

This cross-sectional community-based national survey was conducted through face-to-face interviews (in low-threshold and addiction care services) and online questionnaires (on https://psychoactif.org and other websites). Among the 557 participants, we selected those who were eligible for IV buprenorphine treatment (history of oral OMT, regular opioid injection) (*n* = 371). We used regression models to study factors associated with willingness to receive IV buprenorphine treatment among those with data on willingness (*n* = 353). In those who were willing (*n* = 294), we subsequently studied their willingness to receive daily supervised IV buprenorphine treatment.

**Results:**

Among the selected 353 participants, 59% mainly injected buprenorphine, 15% heroin, 16% morphine sulfate and 10% other opioids. Eighty-three percent of the sample reported willingness to receive IV buprenorphine treatment. Factors associated with willingness were: more than 5 injection-related complications, regular buprenorphine injection, no lifetime overdose, and completion of the questionnaire online. Factors associated with unwillingness to receive daily supervised treatment were younger age (OR[IC95%]=1.04[1.01; 1.07]) and stable housing (OR[IC95%]=0.61[0.37;1.01]) while regular heroin injectors were more willing to receive daily supervision (OR[IC95%]=2.94 [1.42; 6.10]).

**Conclusions:**

PWID were very willing to receive intravenous buprenorphine as a treatment, especially those with multiple injection-related complications. In addition, our findings show that IV buprenorphine may be less acceptable to PWID who inject morphine sulfate. Young PWID and those with stable housing were unwilling to receive IV buprenorphine if daily supervision were required. This preliminary study provides useful information for the development of a clinical trial on IV buprenorphine treatment.

## Strengths and limitations of this study

- To our knowledge, this is the first study to explore the willingness for intravenous buprenorphine treatment among people who inject drugs (PWID).
- We used a community-based approach.
- This study aimed to identify the most appropriate conditions for the introduction of the first injectable treatment for opioid dependence in France.
- Our findings help provide a greater understanding of PWID sub-groups would not be interested in IV buprenorphine treatment and of the relevance of take-home doses for specific sub-groups.
- The study sample is not representative of all PWID, only those who frequently visit harm reduction services and associated websites.

## Background

Despite access to Opioid Maintenance Treatment (oral methadone and buprenorphine) in France, some people who use drugs (PWUD) continue to inject opioids, mainly dissolved buprenorphine tablets [1]. While access to buprenorphine in primary care as an oral opioid maintenance treatment (OMT) has been possible since 1996 thanks to its safety profile [2, 3], methadone induction in France is possible only in specialized care [4]. However, due to its easier accessibility and its comparable efficacy as regards opioid dependence [5], oral buprenorphine is diverted more than methadone through “doctor shopping” practices [6]. Moreover, western countries are facing the growing problem of diversion of prescription opioids (PO) through injection [7]. In France, this is especially true for oral buprenorphine [1]. Morphine sulfate, another prescription opioid (PO) used as an analgesic for persistent pain, is also widely prescribed for opioid dependence and is also diverted by intravenous use [8].

The injection of illicit opioids or PO in a non-medical context has dramatic health consequences for people who inject drugs (PWID). The first is that it can promote the transmission of HIV and Hepatitis [9]. The second is that it can create complications at the injection site [10]. Some studies have reported local lesions associated with the injection of buprenorphine tablets [11] and morphine sulfate capsules [12], but also cardiovascular and pulmonary complications arising from the injection of drugs intended for oral use [13–15]. The use of prescribed injectable treatments for opioid dependence could help promote education about safer injection and reduce risks [16].

In recent years, several studies have tested injectable opioid treatment based on diacetylmorphine - also described as heroin-assisted treatment or medical heroin - and highlighted its effectiveness in terms of increased retention and reduced illicit opioid use [17–20]. Heroin-assisted treatment exists in several countries, including Switzerland, Germany, the Netherlands and Denmark, and has shown positive long-term outcomes such as reduced illicit drug use and criminality, as well as an improved physical, mental and social health [21]. However, this treatment necessitates traveling up to three times a day to the clinic to receive doses [22]. Heroin-assisted treatment is not available in France. As a first step in deciding on whether to implement heroin-assisted treatment, and how to best meet the needs of people who inject buprenorphine illegally, the Inserm collective expert report on Harm Reduction in 2010 [23] suggested evaluating the feasibility of prescribing IV buprenorphine as a treatment.

Buprenorphine is associated with a lower risk of overdose than other full agonist analgesics (diacetylmorphine, morphine sulfate and methadone) [3] as it causes limited respiratory depression, and has a ceiling effect due to its partial agonist profile [24]. This safer profile may translate into less restrictive medical follow-up for stabilized patients. Following up on the report from 2010 and given the current French situation, in 2015 the French medical community and some of the country’s national authorities opened the way for the implementation of a clinical trial to evaluate the effectiveness of IV buprenorphine for opioid-dependent individuals refractory to oral OMT. It must be underlined that IV buprenorphine does not exist as a treatment for opioid-dependent individuals anywhere in the world. In order to inform decisions regarding the protocol for this clinical trial, it was decided to first implement a community-based survey among frequent opioid injectors already exposed to an oral treatment, to identify their willingness to receive IV buprenorphine treatment.

## Methods

### Study design

This cross-sectional community-based survey was conducted in collaboration with the association AIDES and with the support of other associations (Psychoactif, Fédération Addiction, ASUD, Médecins du Monde) in contact with PWID. It was implemented between May and August 2015 throughout France and enrolled PWID in two different ways: a) face-to-face questionnaires proposed in different sites (low-threshold services (needle exchange programs, NGOs) and addiction care services) and b) online questionnaires via the community website Psychoactif.org and via an online link provided to PWID when visiting addiction care services). As the main sites of recruitment were low-threshold services, which principally attract PWID with a low socioeconomic status, to ensure representativeness, it was important to also recruit PWID with a higher status online through Psychoactif.org. As the survey was community-based, PWID and people working with them were involved in its preparation, especially with regard to improving the relevance and wording of the associated questionnaire items. The study received authorization from the national French Data Protection Authority (CNIL).

### Study sample

**Fig. 1:**
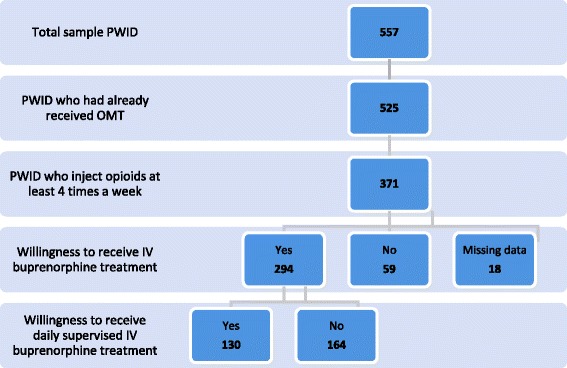
Flow chart – PrebupIV study (*n* = 557). OMT = Opioid Maintenance Treatment

Participation was proposed to all PWID either directly by field stakeholders or through an advertisement on the Psychoactif website for drug users. The survey aimed to target frequent injectors already exposed to OMT. Of the total 557 participants who completed the questionnaire, we excluded those who had no lifetime history of OMT (*n* = 32). We also excluded people who injected opioids fewer than 4 times a week (*n* = 154) (Fig. 1). This cut-off is in line with studies on injectable heroin whose criteria for study inclusion ranged from opioid use in ≥50% of days during the preceding 3 months [18] to daily opioid use [19].

### Questionnaire and variables

A short 31-item questionnaire was administered either online or face-to-face by a community front-line worker. It included 3 sections: 1) socio-demographic characteristics, behavioral and health data (type of OMT, HIV/HCV/HBV status, etc.); 2) drug use practices (type, dose, frequency, polydrug use, etc.), reasons for using drugs by injection, perceived complications; 3) willingness to receive IV buprenorphine treatment, preferences for the type of injecting system (simple vial with syringe or pre-filled syringe) and doses.

The variable “opioid most injected” was built using the number of days per month and identifying the most frequently injected opioid.

Reasons for using drugs by injection were classified into 3 categories as follows: “to avoid withdrawal or to feel capable of daily functioning”, “to get high”, and “for the pleasure of the act of injecting”.

Willingness was assessed using two questions: 1) Are you willing to receive IV Buprenorphine (yes, no)? 2) How would you rate your willingness to receive IV Buprenorphine on a scale from 0 to 10? We used the first question to classify willingness (Yes or No). If the participant answered “No” or “Don’t know” to the first question but had a score ≥ 1 for the second question, willingness was reclassified as positive. If the participant answered “Yes” or “Don’t know” to the first question but had a score = 0 for the second question, then willingness was reclassified as negative.

### Statistical analyses

#### Description of participants eligible for IV buprenorphine

We compared participants included in the analyses with those excluded using a Chi-square or exact Fisher test for discrete variables, and Student’s T test for continuous variables. We also compared participants who completed the questionnaire online with those who answered it face-to-face.

#### Factors associated with willingness to receive IV buprenorphine treatment

We first studied factors associated with willingness to receive IV buprenorphine treatment in all opioid-injecting users of the study sample with available data (*n* = 353). For this sub-group, we then studied factors associated with willingness to receive daily supervised IV buprenorphine treatment (*n* = 294). For both analyses, we used a logistic regression model. We used a threshold of *P*-value <0.20 in the univariate analyses to identify variables eligible to enter the multiple logistic regression model. A backward procedure was then used to select the explanatory variables for the final model, with a *P*-value <0.05.

## Results

### Descriptive analysis of the study sample (*n* = 371)

The only differences found between participants excluded from the analyses and the study sample (*n* = 371) were that the former were more likely to have completed the questionnaire online and to be unemployed.

The comparison between participants who completed questionnaires online (*n* = 95) with those who answered it face-to-face (*n* = 176) showed that the former were more likely to be women, younger, employed, were less likely to use cocaine and alcohol, and were less likely to report being HCV positive (Table 1). Among the 371 included participants, 20% were female and median[IQR] age was 33[28; 40] years. More than half (58%) had stable housing and 32% were employed. With respect to opioid use, 58% mainly injected oral buprenorphine, 15% heroin, 17% oral morphine sulfate and 10% other prescription opiates (methadone, oxycodone, codeine and others). With respect to non-opioid drug use, 50% reported using cocaine, 32% benzodiazepines and 43% alcohol. When asked about the main reason why they injected drugs, 24% answered to get high, 58% to avoid withdrawal symptoms or to feel capable of daily functioning, and 18% for the pleasure of the act of injecting. In morphine sulfate injectors, 42% reported that their main reason for injection was to get high, 50% to avoid withdrawal and 8% the pleasure of injecting. Only 15% of buprenorphine injectors reported that getting high was their main reason for injecting, 61% reporting that it was to avoid withdrawal, and 23% for the pleasure of the act. In heroin injectors, 31% answered to get high, 63% to avoid withdrawal symptoms, and 6% for the pleasure of the act of injecting, while for other opioid injectors, these values were, respectively, 45%, 38% and 17%.

**Table 1:** Descriptive analysis of the eligible participants for injectable treatment in the prebupIV survey (*n* = 371 participants)

	Questionnaire online (*n* = 95)	Questionnaire face-to-face (*n* = 276)	*P*-value	Total (*n* = 371)
Gender			0.03	
male	66 (72)	226 (82)		292 (80)
female	26 (28)	48 (18)		74 (20)
Age – years^a^				
Median [IQR]	31 [24; 40]	34 [29; 40]	0.02	33 [28; 40]
Stable housing			0.61	
No	37 (39)	116 (42)		153 (42)
Yes	57 (61)	158 (58)		215 (58)
Employment			0.02	
No	53 (58)	196 (72)		249 (68)
Yes	38 (42)	77 (28)		115 (32)
Opioid most injected^b^			0.17	
Morphine sulfate	14 (15)	50 (18)		64 (17)
Heroin	21 (22)	35 (13)		56 (15)
Buprenorphine^c^	52 (55)	164 (59)		216 (58)
Other prescription opiates^d^	8 (8)	27 (10)		35 (10)
Main reason for injecting			0.27	
To get “high”	19 (20)	53 (27)		72 (24)
To avoid withdrawal or to feel good enough for daily functioning	60 (65)	109 (55)		169 (58)
Pleasure of the act	14 (15)	38 (19)		52 (18)
Cocaine use^b^			< 0.001	
No	67 (72)	116 (42)		183 (50)
Yes	26 (28)	157 (58)		183 (50)
Benzodiazepine use^b^			0.223	
No	68 (73)	181 (66)		249 (68)
Yes	25 (27)	92 (34)		117 (32)
Alcohol consumption^b^			< 0.001	
No	71 (75)	141 (51)		212 (57)
Yes	24 (25)	135 (49)		159 (43)
Injection-related complications			0.84	
≤ 5 complications	81 (85)	233 (84)		314 (85)
> 5 complications	14 (15)	43 (16)		57 (15)
History of overdose			0.89	
No	74 (78)	213 (77)		287 (77)
Yes	21 (22)	63 (23)		84 (23)
Currently on OMT			0.43	
No	20 (21)	53 (19)		73 (20)
Yes	75 (79)	223 (81)		298 (80)
Self-reported HCV status			0.020	
No	70 (79)	170 (65)		240 (69)
Yes	19 (21)	90 (35)		109 (31)

*IQR* Interquartile range, *OMT* Opioid Maintenance Treatment

^a^in years;

^b^during the previous 12 months;

^c^among buprenorphine injectors: median [IQR] amounts (milligrams) injected per day = 12 [8–16] and median [IQR] numbers of injection per day = 3 [2–4]

^d^methadone, oxycodone, codeine and others

Among buprenorphine injectors, the median[IQR] dose of injected oral buprenorphine was 12[8–16]mg, and the median[IQR] number of buprenorphine injections was 3 [2–4] per day (Table 1).

With respect to complications associated with drug injection, the 5 most frequent complications were hand swelling (17%), vein obstruction (16%), rolling veins (16%), cotton fever (15%) and cutaneous abscesses (14%). Fifteen percent of participants reported more than 5 complications (from 0 to 10). A history of overdose and incarceration were reported by 23% and 40% of PWID, respectively. Eighty percent of the study sample reported they were currently on OMT and 31% reported being HCV positive. Seventy-one percent were on buprenorphine, 10% on methadone, 13% on morphine sulfate, and 6% reported receiving more than one OMT during the previous month. Finally, a high proportion (83%) of the sample answered that they would be willing to use IV buprenorphine as a treatment.. When asked about their preference regarding the type of injecting system, 62% stated they would prefer a simple vial, and 25% a pre-filled syringe, while 13% said they did not know.

### Factors associated with willingness to receive IV buprenorphine treatment

Among those who had data on willingness (*n* = 353), univariate analyses (Table 2) showed no difference in socio-demographic factors between those willing and those not willing to receive intravenous buprenorphine as a treatment for dependence. With respect to drug use practices, results showed that buprenorphine injecting drug users were more likely to accept buprenorphine as an injectable treatment than morphine sulfate (OR [95% CI]: 0.07 [0.03; 0.16]; *p* < 0,001) or heroin injecting drug users (OR [95% CI]: 0.13 [0.05; 0.32]; *p* < 0,001). The variable “reason for injecting drugs” was eligible for the multiple logistic regression analysis, since participants who reported injecting drugs for the pleasure of the act (OR [95% CI]: 5.11 [1.80; 14.49], *p* = 0.002) and to avoid withdrawal (OR [95% CI]: 6.10 [2.89; 12.89]; *p* < 0.001) were more likely to accept IV buprenorphine treatment. Those who reported alcohol consumption were less willing to receive injectable treatment (OR [95% CI]: 0.46 [0.26; 0.81]; *p* = 0.007). Furthermore, reporting more than 5 complications related to drug injection was associated with greater willingness to receive the treatment. A history of overdose during one’s lifetime was associated with lower willingness. Finally, participants on opioid maintenance treatment (OMT) were more likely to accept IV buprenorphine treatment than those not on OMT.

**Table 2:** Factors independently associated with willingness to receive intravenous buprenorphine treatment in the study sample; univariate and multiple logistic regression analyses with OR estimates based on logistic regression analyses (*n* = 353 participants)

			Univariate analysis			Multiple logistic regression		
	No willingness	Willingness^ab^						
	*N* (%)	*N* (%)	OR	[95CI%]	*p*-value	AOR	[95CI%]	*p*-value
Questionnaire								
Questionnaire online	5 (8)	82 (28)	1			1		
Questionnaire face-to-face	54 (92)	212 (72)	0.24	[0.09; 0.62]	0.003	0.16	[0.06; 0.44]	< 0.001
Gender								
Male	46 (78)	233 (80)	1					
Female	13 (22)	58 (20)	0.88	[0.45; 1.74]	0.714			
Age – years^c^								
Median [IQR]	33.5 [30–42]	33 [28–40]	0.99	[0.96; 1.02]	0.624			
Stable housing								
No	23 (40)	122 (42)	1					
Yes	35 (60)	171 (58)	0.92	[0.52; 1.64]	0.779			
Employment								
No	39 (67)	202 (70)	1					
Yes	19 (33)	86 (30)	0.87	[0.48; 1.60]	0.662			
Experience of incarceration								
No	31 (53)	177 (61)	1					
Yes	27 (47)	111 (39)	0.72	[0.41; 1.27]	0.257			
Opioid consumed most^d^								
Buprenorphine	9 (15)	198 (67)	1			1		
Heroin	14 (24)	40 (14)	0.13	[0.05; 0.32]	< 0.001	0.11	[0.04; 0.29]	< 0.001
Prescription Opiates^e^	36 (61)	56 (19)	0.07	[0.03; 0.16]	< 0.001	0.06	[0.03; 0.14]	< 0.001
Duration of opioid use^c^								
Median [IQR]	7 [3–13]	7 [3–10]	0.99	[0.95; 1.03]	0.562			
Main reason for injecting								
To get “high”	25 (58)	46 (19)	1					
To avoid withdrawal symptoms or to feel good enough for daily functioning	13 (30)	146 (61)	6.10	[2.89; 12.89]	< 0.001			
Pleasure of the act	5 (12)	47 (20)	5.11	[1.80; 14.49]	0.002			
Other non-opioid drugs used^d^								
No	10 (17)	67 (23)	1					
Yes	49 (83)	224 (77)	0.68	[0.33; 1.42]	0.307			
Alcohol consumption^d^								
No	24 (41)	176 (60)	1					
Yes	35 (59)	118 (40)	0.46	[0.26; 0.81]	0.007			
Injection-related complications (0–10)								
≤ 5 complications	53 (90)	244 (83)	1			1		
> 5 complications	6 (10)	50 (17)	1.81	[0.74; 4.44]	0.195	3.30	[1.13; 9.61]	0.029
History of overdose								
No	35 (59)	236 (80)	1			1		
Yes	24 (41)	58 (20)	0.36	[0.20; 0.65]	0.001	0.28	[0.14; 0.59]	0.001
Currently on OMT								
No	19 (32)	49 (17)	1					
Yes	40 (68)	245 (83)	2.38	[1.27; 4.44]	0.007			
Self-reported HCV status								
No	35 (64)	192 (70)	1					
Yes	20 (36)	84 (30)	0.77	[0.42; 1.40]	0.388			

*OR* Odds ratio, *CI* Confidence interval

^a^Number of participants willing to receive IV buprenorphine = 294 (83%)

^b^Preference regarding the type of injecting system: simple vial = 62%, pre-filled syringe = 25%, do not know = 13%

^c^in years;

^d^during the previous 12 months;

^e^morphine sulfate, methadone, oxycodone, codeine and others

After adjustment for how the questionnaire was completed (i.e., online versus face-to-face), multiple logistic regression analysis (Table 2) showed that those who regularly injected buprenorphine (heroin: AOR [95% CI]: 0.11 [0.04; 0.29], *p* < 0.001; prescription opiates^1^: AOR [95% CI]: 0.06 [0.03; 0.14], *p* < 0.001) and those who reported a large number of associated complications (> 5) (AOR [95% CI]: 3.30 [1.13; 9.61], *p* = 0.029) were more likely to be willing to receive IV buprenorphine treatment for opioid dependence, while those who had a history of overdose (AOR [95% CI]: 0.28 [0.14; 0.59], *p* = 0.001) were less likely.

### Factors associated with willingness to receive daily supervision of IV buprenorphine treatment

Among those who were willing to receive IV buprenorphine treatment (*n* = 294), those who rejected the possibility of daily supervision were more often younger (AOR [95% CI]: 1.04 [1.01; 1.07], *p* = 0.014) and had stable housing (AOR [95% CI]: 0.61 [0.37; 1.01], *p* = 0.053). On the contrary, heroin injectors (AOR [95% CI]: 2.94 [1.42; 6.10], *p* = 0.004) were more willing to receive daily supervision (Table 3).

**Table 3:** Factors independently associated with willingness to receive daily supervised buprenorphine injection at the medical center; univariate and multiple logistic regression analyses with OR estimates based on logistic regression analyses (*n* = 294 participants)

			Univariate analysis			Multiple logistic regression analysis		
	No willingness	Willingness^a^						
	N (%)	N (%)	OR	[95CI%]	*p*-value	AOR	[95CI%]	*p*-value
Questionnaire								
Questionnaire online	50 (30)	32 (25)	1					
Questionnaire face-to-face	114 (70)	98 (75)	1.34	[0.80; 2.26]	0.266			
Gender								
Male	125 (77)	108 (84)	1					
Female	37 (23)	21 (16)	0.66	[0.36; 1.19]	0.166			
Age – years^b^								
Median [IQR]	32 [27–38]	34 [28–41]	1.02	[1.00; 1.05]	0.087	1.04	[1.01; 1.07]	0.014
Stable housing								
No	61 (37)	61 (47)	1			1		
Yes	103 (63)	68 (53)	0.66	[0.41; 1.05]	0.083	0.61	[0.37; 1.01]	0.053
Employment								
No	115 (71)	87 (69)	1					
Yes	47 (29)	39 (30)	1.10	[0.66; 1.82]	0.721			
Experience of incarceration								
No	103 (64)	74 (58)	1					
Yes	58 (36)	53 (42)	1.27	[0.79; 2.05]	0.323			
Opioid most injected^c^								
Buprenorphine	121 (74)	77 (59)	1			1		
Heroin	15 (9)	25 (19)	2.62	[1.30; 5.28]	0.007	2.94	[1.42; 6.10]	0.004
Prescription opiates^d^	28 (17)	28 (22)	1.57	[0.87; 2.85]	0.138	1.52	[0.83; 2.76]	0.173
Duration of opioid use^b^								
Median [IQR]	7 [4–10]	6 [3–11]	1.00	[0.96; 1.04]	0.963			
Main reason for injecting								
To get “high”	24 (17)	22 (22)	1					
To avoid withdrawal symptoms or to feel good enough for daily functioning	83 (59)	63 (64)	0.83	[0.43; 1.61]	0.578			
Pleasure of the act	33 (24)	14 (14)	0.46	[0.20; 1.08]	0.076			
Other non-opioid drugs used^c^								
No	39 (24)	28 (22)	1					
Yes	124 (76)	100 (78)	1.12	[0.65; 1.95]	0.680			
Alcohol consumption^c^								
No	102 (62)	74 (57)	1					
Yes	62 (38)	56 (43)	1.24	[0.78; 1.99]	0.360			
Injection-related complications (0–10)								
≤ 5 complications	139 (85)	105 (81)	1					
> 5 complications	25 (15)	25 (19)	1.32	[0.72; 2.44]	0.367			
History of overdose								
No	139 (85)	97 (75)	1					
Yes	25 (15)	33 (25)	1.89	[1.06; 3.38]	0.031			
Currently on OMT								
No	25 (15)	24 (18)	1					
Yes	139 (85)	106 (82)	0.79	[0.43; 1.47]	0.463			
Self-reported HCV status								
No	106 (69)	86 (70)	1					
Yes	47 (31)	37 (30)	0.97	[0.58; 1.63]	0.909			

*OR* Odds ratio, *CI* Confidence interval, *IQR* Interquartile range, *OMT* Opioid maintenance treatment

^a^Number of participants willing to receive daily supervised IV buprenorphine = 130 (44%);

^b^in years;

^c^during the previous 12 months;

^d^ morphine sulfate, methadone, oxycodone, codeine and others

## Discussion

To our knowledge, this is the first study to explore drug users’ willingness to receive a novel injectable treatment for opioid dependence: intravenous buprenorphine. The results of this preliminary community-based survey clearly show that the level of willingness is high and provide a strong argument for the development of intravenous buprenorphine treatment. Not all the injecting opioid users who participated stated they would engage in this treatment however. Specifically, buprenorphine injectors indicated they would be more willing to participate than morphine sulfate injectors and heroin injectors. This difference between buprenorphine and heroin or morphine sulfate injectors is not surprising, as the effect and consequences of each substance are very different [25], as some PWUD report searching for a rewarding effect with morphine sulfate use [26]. This is corroborated by our results which show that 42% of morphine sulfate injectors wanted to get “high” compared with only 15% of buprenorphine users.

Our findings show that 23% of buprenorphine injectors did so for the pleasure of the act, while only 8% of morphine sulfate injectors gave this reason. Heroin users were also less willing to receive IV buprenorphine than buprenorphine users, but the difference was less marked than for morphine sulfate users.

Another interesting result is that individuals with a greater number of complications (>5) were more likely to accept treatment with intravenous buprenorphine. It has been shown that almost half of PWID hospitalizations are due to cutaneous injection-related infections [27].

In addition, PWID with a history of overdose were less likely to accept IV buprenorphine. A recent study on PWID in a Canadian setting showed that the risk of overdose is higher in those who use both heroin and prescription opioids than in exclusive PO injectors [7]. Individuals with a history of overdose are more likely to have used full agonists more frequently (and reduced use of buprenorphine) and may not be willing to exclusively use injectable buprenorphine or to engage in related care. A history of overdose may be a proxy of longer history opioid use and dependence and difficult socioeconomic context (social vulnerability, prison experience, etc). Engaging these patients in care remains a challenge, but other tools like safer injecting facilities [28, 29] or supervised heroin programs can be a first entry point [21]. Finally, those who completed the questionnaire online were more willing to receive IV buprenorphine treatment. This may be because it was less comfortable for those who answered the questionnaire face-to-face to admit that they needed intravenous treatment [30].

Our findings also show also that not all those willing to receive IV buprenorphine would accept daily supervised injection in a medical center. More specifically, those less willing to receive daily supervision were more often younger and had stable housing. This highlights the importance of take-home doses for stabilized patients.

Heroin users willing to receive IV buprenorphine treatment were more likely to accept daily supervision. This suggests that this sample (with previous experience of OMT) may constitute suitable candidates for intravenous supervised buprenorphine treatment. More generally, our results suggest the need to diversify therapeutic options for PWID and to envisage the inclusion of supervised injectable diacetylmorphine [21] for those injecting full-agonist PO.

Some limitations have to be acknowledged. First, it is known that self-reports are subject to social desirability bias. However, their reliability in drug-using populations has been demonstrated [31]. Second, not all the survey items used were from validated questionnaires. However, they were validated here by scientific knowledge and community-based stakeholders. In terms of sociodemographic profile, our study sample was similar to those in related studies except for employment, with our study reporting a higher rate [17, 18]. In addition, the sample of PWID participating in this survey may not be representative of all PWID in France. Nevertheless, our sample comprised PWID who frequently visit harm reduction services and associated websites, and accordingly are probably more informed than others about the availability of new treatments.

The promising findings from this preliminary study regarding PWID willingness to receive injectable buprenorphine treatment for opioid dependence are also important to obtain French institutional authorization to develop the protocol for the forthcoming clinical trial. There are two main arguments that can justify the use of injectable buprenorphine as a treatment: the first is lower abuse liability of buprenorphine than full-opioid agonists [32]. The second is that injectable buprenorphine has fewer complications than injecting oral buprenorphine [11, 33].

## Conclusions

To conclude, the present results confirm that not all injecting drug users would be interested in IV buprenorphine treatment. Some heroin injectors, those who divert oral buprenorphine, and those with multiple injection-related complications would appear to be the most suitable populations to propose this treatment to, as part of their medical follow-up. Our work opens the way for a clinical trial which will evaluate the most appropriate conditions for the introduction of the first injectable treatment for opioid dependence in France. In countries where an opioid overdose crisis exists, IV buprenorphine may be a relevant therapeutic option for PWID [34]. In countries where IV diacetylmorphine is already available [35], IV buprenorphine may be a complementary therapeutic option for those who need less supervised treatment. Indeed, the medical follow-up envisaged for IV buprenorphine prescription is less restrictive than that for existing follow-up for treatment with injectable heroin as buprenorphine has a safer profile. This fact should enable physicians to prescribe take-home doses and to recommend less supervised injection in medical centers, according to the risk profile of the individual patient.

## Abbreviations

AOR: Adjusted odds ratio
CI: Confidence interval
CNIL: Commission Nationale de l’Informatique et des Libertés / French data protection authority
HBV: Hepatitis B virus
HCV: Hepatitis C virus
HIV: Human immunodeficiency virus
INSERM: Institut National de la Santé et de la Recherche Médicale
IQR: Interquartile range
IRD: Institut de Recherche pour le Développement
IV: Intravenous
OMT: Opioid maintenance treatment
OR: Odds ratio
ORS PACA: Observatoire Régionale de la Santé Provence-Alpes-Côte d’Azur
PO: Prescription opioid
PWID: People who inject drugs
PWUD: People who use drugs
SESSTIM: Sciences Economiques and Sociales de la Santé and Traitement de l’Information Médicale
UMR: Unité Mixte de Recherche

## Footnotes

1 morphine sulfate, methadone, oxycodone, codeine and others
